# A Rare t(3;15;17) in a Patient with Acute Promyelocytic Leukemia: Case Report and Review of the Literature

**DOI:** 10.3390/diagnostics15151901

**Published:** 2025-07-29

**Authors:** Linda Shi, Chu En Chen, Tahmeena Ahmed, Jacob Rocha, Pons Materum, Sashank Cherukuri, Leah Gallagher, Paula Fernicola, Roxana Ponce, Htien Lee, Christina Giordano, Gabriela Evans, Changtai Tian, Carlos A. Tirado

**Affiliations:** 1 The International Circle of Genetics Studies Project, New York Chapter, 25 Pinnacle Dr., New York, NY 11777, USA; linda.shi@stonybrook.edu (L.S.); chuen.chen@stonybrook.edu (C.E.C.); 2 Renaissance School of Medicine, Stony Brook University, Stony Brook, NY 11794, USA; tahmeena.ahmed@stonybrookmedicine.edu; 3 Department of Pathology, Renaissance School of Medicine, Stony Brook University, Stony Brook, NY 11794, USA; sashank.cherukuri@stonybrookmedicine.edu; 4 The Cytogenetics Lab at the Department of Pathology, Stony Brook University Hospital, Stony Brook, NY 11794, USA; jacob.rocha@stonybrookmedicine.edu (J.R.); pons.materum@stonybrookmedicine.edu (P.M.); leah.gallagher@stonybrookmedicine.edu (L.G.); paula.fernicola@stonybrookmedicine.edu (P.F.); roxana.ponce@stonybrookmedicine.edu (R.P.); htien.lee@stonybrookmedicine.edu (H.L.); christina.giordano@stonybrookmedicine.edu (C.G.); gabriela.evans@stonybrookmedicine.edu (G.E.); changtai.tian@stonybrookmedicine.edu (C.T.)

**Keywords:** acute promyelocytic leukemia, cytogenetics, *PML::RARA*, atypical karyotype

## Abstract

We present a 48-year-old female with a past medical history of endometrioid adenocarcinoma who presented with symptoms of spontaneous gum bleeding, post-coital bleeding, and upper extremities–lower extremities-abdomen ecchymosis. Initial laboratory findings were significant for cytopenia and disseminated intravascular coagulation (DIC). Due to a suspected case of acute promyelocytic leukemia (APL), conventional karyotyping and fluorescence in situ hybridization (FISH) were performed. FISH analysis confirmed an unusual chromosome rearrangement that affected chromosomes 3, 15, and 17. This t(3;15;17)(q29;q24;q21) was characterized by the presence of *PML::RARA* fusion on the derivative chromosome 15. Treatment at the hospital with standard APL therapy of all-trans retinoic acid (ATRA) and arsenic trioxide (ATO) was complicated by the development of differentiation syndrome, which necessitated the temporary stoppage of ATO. However, complete remission was achieved despite complications after starting consolidation treatment.

## 1. Introduction

Acute promyelocytic leukemia (APL) is a unique subtype of acute myeloid leukemia (AML) marked by the uncontrolled proliferation of promyelocytes [1]. It is specifically characterized by the formation of an abnormal fusion gene, *PML::RARA,* as noted in the 2022 WHO Classification of AMLs [2]. The fusion gene blocks and stops myeloid cells from differentiating beyond the promyelocyte stage, resulting in an abnormal accumulation of promyelocytes in the blood and bone marrow [1,3]. Common symptoms include anemia, bleeding/hemorrhagic complications (gingivitis, nosebleeds, heavy menstruation, intracranial hemorrhage, GI bleeding), frequent bruising, weakness, ecchymosis, asthenia, and easy fatigue, etc. [3]. APL constitutes 7–8% of all AML cases with approximately 600–800 new cases diagnosed per year in the USA [4]. On a global scale, it is reported that Mexico, Central and South America, Italy, and Spain have higher incidences of APL, with incidence rates especially high in Hispanic children and young adults [1,5,6]. Currently, APL is considered the most curable type of adult leukemia, with a cure rate of 90–95% [7,8].

The cells of an APL patient are typically characterized by a chromosomal abnormality involving the translocation of chromosomes 15 and 17, t(15;17)(q24;q21), which results in the *PML::RARA* fusion gene. The *RARA* (retinoic acid receptor α) gene is located on chromosome 17q21.2 and it provides instructions for making a transcription factor known as the retinoic acid receptor alpha (RARα). RARα regulates the transcription of genes involved in the maturation of promyelocytes. The *PML* gene is located on chromosome 15q24.1 and it makes tumor suppressor PML proteins that prevent uncontrolled cell proliferation [9,10]. Abnormal *PML::RARA* fusion gene and its protein products bind to DNA and repress gene expression as usual, but it does not respond to signals to induce gene transcription. As a mutated fusion gene, the *PML::RARA* protein products allow uncontrolled cell proliferation and block the differentiation of promyelocytes, unlike normal PML and RARα products.

Typical cases (>95%) of APL involve a balanced, two-way translocation of chromosomes 15 and 17, but there have been increasing reports of complex atypical karyotypes. Variant rearrangement of APL includes three-way translocations involving another chromosome in addition to the hallmark chromosomes of 15 and 17. These variant translocations are usually cryptic and will require further FISH and other molecular tests to confirm. In this case study, we present a case with the atypical chromosomal rearrangement of t(3;15;17) with *PML::RARA* fusion.

## 2. Case Presentation

The patient is a 48-year-old female with a past medical history of endometrioid adenocarcinoma treated with hysterectomy and bilateral salpingo-oophorectomy, morbid obesity status-post bypass surgery, and anxiety. The patient was initially presented to Stony Brook University Hospital with persistent cephalalgia, spontaneous gingival hemorrhage, post-coital vaginal bleeding, and ecchymosis of the lower and upper extremities as well as the abdominal region. CT head revealed acute or chronic subdural hemorrhage. CBC was significant for 33% blasts, WBC: 8.5 K/µL, Hemoglobin: 9.8 g/dL, Platelets: 12 K/µL, LDH: 618 IU/L, INR: 1.4, PTT: 29.7 s, Fibrinogen: 196 mg/dL, D dimer: 7414 ng/mL, and Haptoglobin: 137 mg/dL (Table 1).

**Figure 1:**
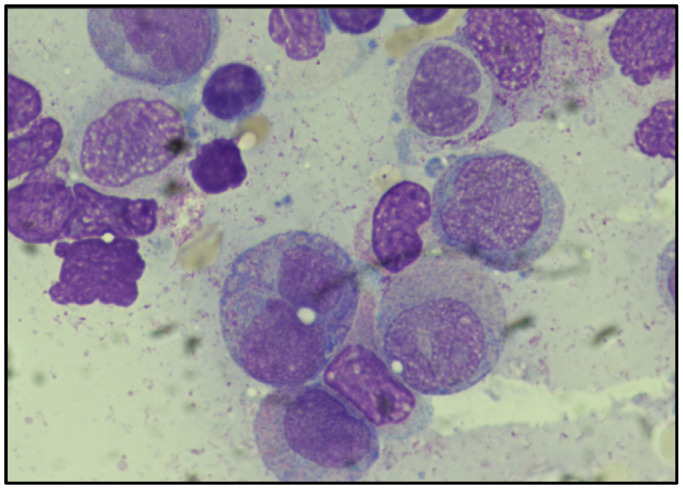
Large size blasts with abundant cytoplasm and open chromatin, occasional nuclear indentation, and cytoplasmic granules with rare Auer rods suspicious for APL.

The peripheral blood smear was significant for blasts with granules (Figure 1). Flow cytometry demonstrated an abnormal population of myeloid blasts. Diffuse intravascular coagulation secondary to acute promyelocytic leukemia was suspected. A bone marrow biopsy was performed and the diagnosis of acute promyelocytic leukemia with *PML::RARA* fusion was rendered.

The subdural hemorrhage was treated with middle meningeal artery embolization and the patient was started on all-trans retinoic acid (ATRA) and arsenic trioxide (ATO). The clinical course was complicated by differentiation syndrome with fevers, ARDS, and QT prolongation, requiring transient cessation of ATO. The patient also developed papilledema and elevated intracranial pressure, requiring transient cessation and tapering up of ATRA. A repeat bone marrow biopsy was performed and showed remission with no morphological or immunophenotypic evidence of residual APL. Flow cytometry and FISH studies at this time were also concurrently negative for APL. The patient was subsequently started upon consolidation treatment with ATO and ATRA.

## 3. Materials and Methods

### 3.1. Conventional Cytogenetics

Chromosome analysis was performed using conventional Cytogenetics protocols. The ISCN was performed using ISCN 2024 [11].

### 3.2. Molecular Cytogenetics

Fluorescence in situ hybridization (FISH) was performed with LSI PML/LSI RARA DC, DF translocation probe (15q22-24,17q21) from Abbott (Des Plaines, IL, USA), and XL RARA BA (17q21.1-21.2) FISH probe from MetaSystems (Medford, MA, USA) as well as the Subtelomre 3p, 3q from CytoCell (Cambridge, CB4 0PZ, UK).

## 4. Results

### 4.1. Conventional Cytogenetics

**Figure 2:**
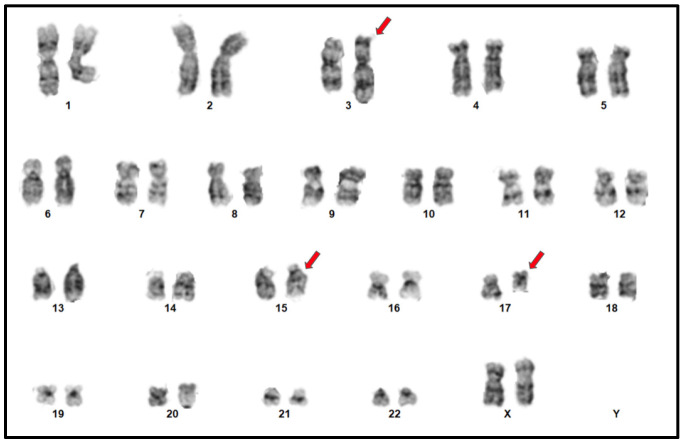
G-Band karyotype analysis with bone marrow. 46,XX,der(3)(3pter→q29::17q21→qter),der(15)(15pter→q22-24::17q21→17qter),der(17)(p13→q21::3q29→qter) in all 20 metaphases. The abnormal chromosomes are indicated by red arrows.

Chromosome analysis of 20 trypsin-Giemsa banded metaphase spreads, 2 from unstimulated blood cultures and 18 from a 24-h culture at a resolution of 400 chromosome bands, showed an abnormal female karyogam with a t(15;17) as well as the involvement of the long arm of chromosome 3 in a three-way translocation with translocations 3 and 17, a derivative chromosome 15 involving a *PML::RARA*, and a derivative chromosome 17 with a translocation between 17q21 and 3q29. This karyotype was described (Figure 2) as the following: 46,XX,der(3)(3pter→q29::17q21→qter),der(15)(15pter→q22-24::17q21→17qter),der(17)(p13→q21::3q29→qter).

### 4.2. Molecular Cytogenetics

**Figure 3:**
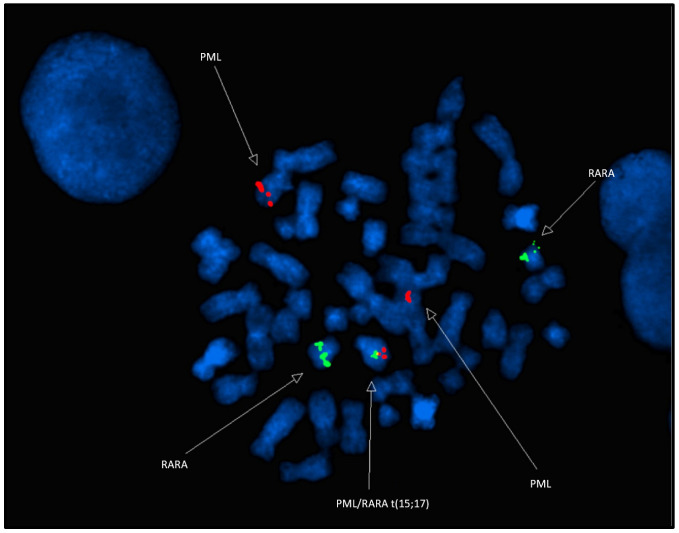
FISH results with the LSI PML/LSI RARA DC, DF translocation probe (15q22-24,17q21) were consistent with an atypical pattern of PML::RARA (2 orange, 2 green, 1 fusion) in 84% (169/200) of the nuclei.

FISH analysis using the LSI PML-RARA dual-color translocation probes showed a *PML::RARA* fusion signal on the derivative chromosome 15, an orange *PML* signal on the normal chromosome 15, a green *RARA* signal on the normal chromosome 17, and a green *RARA* signal on the long arm of chromosome 3 (Figure 3). This is an atypical *PML::RARA* pattern with translocations including chromosomes 3, 15, and 17, leading to a derivative chromosome 3 involving translocations 3 and 17, a derivative chromosome 15 involving a *PML::RARA*, and a derivative chromosome 17 with a translocation between 17q21 and 3q29. These findings were characterized as nuc ish 15q22-24,17q21(PML,RARA)x3(PML con RARAx1) [168/200], 17q21.1-21.2 (5′RARA)x2 (5′RARA con 3′RARAx1) [184/200].

**Figure 4:**
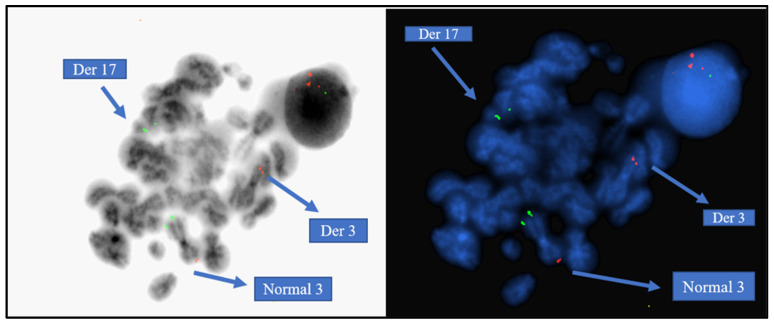
FISH on previously G-banded metaphases using the subtelomere probes for 3p and 3q.

ToTelVysion mix 3 (TelVysion 3p SpectrumGreen, TelVysion 3q SpectrumOrgane, TelVysion 22q SpectrumOrange and SpectrumGreen, LSI BCR (22q11) Spectrum Aqua) by Abbott was used on metaphases to determine the location of the subtelomere 3q. The Abbott LSI BCL6 BAR (3q27) confirmed the subtelomere 3q signal on derivative chromosome 17. The *RARA* break-apart probe was used to confirm the result, where there was a rearrangement of (1 orange, 1 green, and 1 fusion) in 92% of the nuclei. The Abbott LSI BCL6 BAR (3q27) also confirmed the breakpoint on the derivative of chromosome 3 (Figure 4).

In light of the FISH performed, the Karyogram was described as 46,XX,der(3)(3pter→q29::15q22-q24),der(15)(15pter→q22-24::17q21→17qter), der(17)(p13→q21::3q29→qter) [12].ish der(3)(BCL6+,PML+),der(15)(PML::RARA+),der(17)(RARA+,D3S1272+).nuc ish 15q22-24,17q21(PML,RARA)x3(PML con RARAx1) [168/200],17q21.1-21.2(5′RARA,3′RARA)x2(5′RARA con 3′RARAx1) [184/200]

## 5. Discussion

Acute promyelocytic leukemia is characterized by the hallmark translocation between chromosome 15 and 17 or the presence of the *PML::RARA* fusion gene. As a rare subtype of AML, APL only accounts for approximately 7–8% of all total AML cases. The average age of onset for APL is at the median age of 47 years in both males and females [4]. The rate of APL incidences increases with age, with most cases occurring between 75 and 79 years old [13].

Variant and cryptic cases involving another chromosome besides 15 and 17 are known to occur. Around 10% of APL patients are diagnosed with atypical three-way or other complex translocations. Several three-way translocations have been reported in the literature involving chromosomes 1, 2, 3, 4, 6, 8, 11, 19, 20, 22, and X [14].

This case study presents a rare occurrence of atypical APL with a three-way translocation involving chromosome 3. Three translocations have been characterized using conventional karyotyping and FISH analysis. It is as follows: 17q21→qter translocated to 3pter→q29, resulting in a der(3); 17q21→qter translocated to 15pter→q22-24, resulting in the *PML::RARA* fusion; and finally a small piece of subtelomere 3q29→qter translocated to 17p13→q21, resulting in der(17). In cases like this, the PML signal appears on the derivative chromosome 3, and the subtelomere of chromosome 3q was observed on the derivative chromosome 17. The BCL6 probe on 3q27 was still intact on chromosome 3 so our breakpoint on the long arm of chromosome 3 was 3q29, which excludes the subtelomeric region on 3q. These findings rendered a diagnosis of acute promyelocytic leukemia with a three-way translocation involving chromosomes 3q, 15, and 17 and producing a *PML::RARA* fusion on the derivative chromosome 15.

A good prognosis is demonstrated in patients with classical APL with t(15;17) or those with the *PML::RARA* fusion due to the use of all-trans retinoic acid (ATRA) and arsenic trioxide (ATO) [13]. Furthermore, patients will generally demonstrate a favorable prognosis as long as the *PML::RARA* fusion protein is intact and present [14]. The presence of the *PML::RARA* gene is essential to prognosis; therefore, atypical APL patients with the *PML::RARA* fusion gene respond similarly to patients with classical APL to the same standard treatments [15]. This suggests a minimal role of complex chromosomal rearrangements in the prognosis [16]. There is acknowledgeable treatment resistance that remains as a concern despite the addition of ATO in the present day. Patients with APL have the possibility of early mortality, bleeding complications, and developing differentiation syndrome [3]. From previous clinical studies, it was often observed that patients who overcome classic clinical symptoms of APL such as bleeding and coagulation have a greater chance of survival with targeted treatments such as ATRA and ATO [17].

In a review of the literature, there have only been five previous cases of three-way translocation in APL (Table 2) that involve chromosome 3 with classical and atypical t(15;17) rearrangements [12,18,19,20]. These all involved three-way translocations with the short arm of chromosome 3 in breakpoints of 3p21, 3p21, 3p21, and 3p12. Of the five previous cases, there was only one case where the long arm of chromosome 3 was translocated in locus region 3q25 reported by Wang, 2016 [12]. Our current case study is the second case involving a three-way translocation t(3;15;17) in the locus region 3q29.

Bernstein et al. reported early observations of complex chromosomal translocations between chromosomes 15 and 17 found in APL patients [18]. Despite the presence of the typical t(15;17) translocation, these patients fared badly in treatment, which was predictive of the then-limited therapeutic strategies. Similarly, McKinney et al. in 1994 studied *PML* and *RARA* gene rearrangements in APL patients with variant or complex translocations, including cryptic or atypical forms of the typical t(15;17) [19]. The study demonstrated how such rearrangements may occur within cytogenetically complex settings and thus may not always be detected through conventional karyotyping. These cases of t(3;15;17) during the late 20th century led to poor outcomes attributable to the lack of specific molecular diagnostics at the time and the lack of modern targeted therapies such as ATRA and ATO, now standard and highly effective for inducing remission in APL. Freeman et al. reported t(3;17;15)(q27;q21;q22) in 2009 [20]. For effectively detecting these rearrangements, further molecular diagnostic methods, including fluorescence in situ hybridization (FISH) and reverse transcription–polymerase chain reaction (RT-PCR), were needed. These advanced techniques were capable of establishing the presence of the *PML::RARA* fusion gene in cases where standard karyotyping cannot demonstrate the complexity of the chromosomal abnormalities. Throughout this study by Freeman et al., the usual treatment protocols for APL were still evolving, and the use of targeted agents such as all-trans retinoic acid (ATRA) and arsenic trioxide (ATO) was not yet widespread. However, the patient was able to achieve complete remission. Wang et al. described a rare three-way chromosomal translocation involving chromosomes 3, 17, and 15: t(3;17;15)(q25;q21;q24) [12]. A bone marrow study was found with 84.5% atypical promyelocytes, and fluorescence in situ hybridization (FISH) detected the *PML::RARA* fusion gene in 92% of the cells studied. Reverse transcription–polymerase chain reaction (RT-PCR) detected the long-form (L-form) *PML::RARA* transcript but not the reciprocal *PML::RARA* fusion. The patient was initially treated with arsenic trioxide (ATO) monotherapy after intolerance to all-trans retinoic acid (ATRA). The patient, on occasion of promyelocyte differentiation, developed intracranial hypertension signs and leukocytosis. Molecular remission was achieved after target therapy, and consolidation with cytarabine was administered. The patient eventually achieved complete remission. Cytogenetically, our present case report of a 48-year-old woman with acute promyelocytic leukemia (APL) and unusual three-way translocation—46,XX,der(3)(3pter→q29::17q21→qter),der(15)(15pter→q22-24::17q21→17qter), der(17)(p13→q21::3q29→qter) [12]—that varies from the more typical t(15;17)(q22;q21) translocations, such that the breakpoint regions on 15q for *PML* and on 17q for *RARA* are associated with derivative chromosomes that also involve material from 3q29. The *PML* gene at 15q22-24 and *RARA* gene at 17q21 were fused together alongside a three-way translocation of 3q material—a pattern also seen in other complicated translocations by Freeman et al. [20] and Wang et al. [2]. In contrast, earlier cases reported by Bernstein et al. [18] and McKinney et al. [19] identified similar cytogenetic complexity but lacked the technology to completely delineate the breakpoints and lacked the benefit of present-day targeted therapy. Our patient received the contemporary standard of care treatment of both all-trans retinoic acid (ATRA) and arsenic trioxide (ATO) and achieved complete molecular remission. However, the development of differentiation syndrome on therapy—a side effect that occurred in the current century for the first time—is a compromise in modern treatment resulting from the elicited biologic response to differentiation induction.

A few cryptic cases of APL with a two-way translocation involving the long arm of chromosome 3 have also been reported in the literature (Table 3), given that this is a rare occurrence in APL. Appachu et al. reported a rare t(3;15)(q26;q13) translocation without the presence of classical t(15;17) translocation, as identified by standard karyotyping [21]. However, the *PML::RARA* fusion was identified by molecular analysis. The patient, who had a congenital cardiac anomaly that prevented administration of anthracycline, was given arsenic trioxide (ATO) and all-trans retinoic acid (ATRA) and achieved hematologic remission on day 45. Complications included differentiation syndrome and pneumonia, but the patient did respond in the end. Contrastingly, Chen et al. described the detection of a novel RARα fusion gene, *TBLR1-RARα*, in an APL with complex chromosomal aberrations such as t(3;17)(q26;q21) [22]. Unlike typical *PML::RARA* fusion, the patient was resistant to ATRA-based induction but sensitive to ATO monotherapy. Functional assays demonstrated that *TBLR1-RARα* fusion protein, while structurally analogous in some respects to other RARα fusions, recruited more potent transcriptional corepressors and exhibited reduced transcriptional activity. Pharmacologic levels of ATRA, however, were still able to trigger degradation of the fusion protein and trigger differentiation, though clinical sensitivity was reduced. Similarly, Cheng et al. also reported the novel fusion gene *FNDC3B-RARA* with a classic APL presentation, but *PML::RARA* detection tested negative [23]. Cytogenetic analysis revealed a t(3;17)(q26;q21) translocation, and molecular analysis revealed a chimeric *FNDC3B-RARA* protein, the 13th described *RARA* fusion. This chimera preserved the RARA DNA- and ligand-binding regions as well as the FNDC3B fibronectin type III regions. The patient responded to ATRA treatment initially but recurred after eight months with a clonal evolution. Functional analyses demonstrated that the fusion protein suppressed transcription more potently than wild-type *RARA* in the absence of ATRA, but paradoxically also had an ATRA-induced activation response, suggesting a suboptimal therapeutic window. Conversely, our present case most closely resembles the pediatric case reported by Appachu et al. [21] where both the patients were managed with target therapy irrespective of non-classical chromosomal findings. Unlike the *TBLR1-RARα* and *FNDC3B-RARA* cases, which involved novel fusion partners with altered transcriptional dynamics and less predictable therapeutic outcomes, the presence of *PML::RARA* in our present case enabled successful application of standard treatment protocols.

The classic t(15;17)(q24;q21)/*PML::RARA* fusion gene is the predominant pathogenesis of APL with >95% case presentations [24], while the remaining rare variant APL cases involve novel fusions and mutations in the long arm of chromosome 3 including alternative *RARA* or *RARG* gene fusions, such as *TBLR1-RARA, FNDC3B-RARA,* and *TFG-RARA* [24,25]. *TBLR1*, positioned at chromosome 3q26.32, encodes for a hematopoietic stem cell-enriched protein, which participates in both t(3;17)(q26;q21) translocation, as well as a cryptic insertion to form a fusion gene with *RARA*. The *TBLR1::RARA* fusion gene includes exon 5 from *TBLR1* and exon 3 from *RARA*, which can form homo- and hetero-dimers and interacts with transcriptional corepressors to further reduce transcriptional activity [22,25,26]. *FNDC3B* on 3q26 is involved in adipocyte differentiation (located at chromosome 3q26) and can create fusion transcripts with *RARA* via the t(3;17)(q26;q21) translocation along with reciprocal *RARA-FNDC3B* transcripts, which also represses *RARA*′s transcriptional regulation, increases its repressor activity, prevents proper differentiation, and allows for the development of APL. *FNDC3B::RARA* has nuclear localization and can dimerize with itself, *RXRA*, and *FNDC3B* [24,26]. *TFG* is involved in the endoplasmic reticulum and microtubule function and is located at chromosome 3q12. It creates a *TFG::RARA* fusion through a rare t(3;14;17)(q12;q11;q21) translocation to join exon 7 of *TFG* with exon 3 of *RARA* [25,27]. Resistance to ATRA and/or arsenic trioxide was common, and even with the initial remission rate, most patients relapsed in these notable variants. This may indicate a similar mechanism involving a pathogenetically important gene of chromosome 3 in the long arm relevant to our present case. However, there is currently no evidence of pathogenetically important genes on the breakpoint of 3q29 prevalent in our present case. Further molecular studies of complex and variant structural rearrangements may expose additional pathogenetically important genes.

The t(3;15;17) translocation in APL remains unclear in the past literature, given a total of 6 cases reported with similar clinical presentation. FISH analysis of this patient showed only one *PML::RARA* fusion gene.

In conclusion, this case highlights a rare, complex three-way translocation t(3;15;17) in acute promyelocytic leukemia (APL), which results in the *PML::RARA* fusion gene on a derivative chromosome 15. Despite the presence of this atypical karyotype, the patient responded favorably to the current standard treatment regimen of all-trans retinoic acid (ATRA) and arsenic trioxide (ATO), achieving complete remission. This reinforces the importance of the *PML::RARA* fusion as a relevant prognostic and therapeutic marker for APL, regardless of the complexity of its chromosomal rearrangements. As diagnostic technologies advance, the identification and characterization of such rare variants become increasingly feasible, allowing for more tailored and effective clinical management. Future studies should continue to explore the molecular underpinnings of variant translocations in APL to enhance diagnostic accuracy and inform targeted therapeutic strategies.

## Figures and Tables

**Table 1:** Touch prep differentials.

	Patient %	Reference Range %
Blasts + Promyelocytes	89.0	0–3
Myelocytes	2.0	8–17
Metamyelocytes	1.0	10–25
Band/PML Neutrophils	4.0	16–27
Eosinophils	1.0	1–6
Lymphocytes	0.0	10–23
Plasma Cells	0.0	0–2
Monocytes	0.0	0–3
Nucleated RBC	2.0	14–30

**Table 2:** Comparison to other three-way translocations in acute promyelocytic leukemia involving chromosome 3.

Reference	Karyotype	Presence of Classic t(15;17)	Age	Treatment
Bernstein et al., 1980 [18]	46, XY, t(3;15;17)(p21;q25?6?;q21?2?); 4p+	No	N/A	N/A
McKinney et al., 1994 [19]	46, XX, t(3;17;15)(p21;q21;q22) [12]	No	51 y/o	Ara-C, daunorubicin, and trans-retinoic acid
McKinney et al., 1994 [19]	46, XX, t(3;17;15)(p21;q21;q22) [12]	No	63 y/o	Ara-C, daunorubicin, and mitoxantrone
Freeman et al., 2009 [20]	46, XY, t(3;15;17)(p12;q21;q22) [12]	Yes	78 y/o	Idarubicin, Ara-C, and ATRA
Wang et al., 2016 [2]	46, XY, der(3) t(3;17;15)(q25;q21;q24), der(15)t(3;17;15),der(17),der(17)(q11q12)	Yes	33 y/o	ATO, daunorubicin, dexamethasone, intrathecal methotrexate, and cytosine arabinoside * patient was intolerant to ATRA
*Present Case*	46,XX,der(3)(3pter→q29::17q21→qter),der(15)(15pter→q22-24::17q21→17qter),det(17)(p13→q21::3q29→qter)	Yes	48 y/o	All-trans retinoic acid (ATRA) and arsenic trioxide (ATO)

**Table 3:** Comparison to other two-way translocations in acute promyelocytic leukemia involving the long arm of chromosome 3.

Reference	Karyotype	Presence of Classic t(15;17)	Age	Treatment	Outcome
Heim et al., 1988 [21]	t(3;15)(q21;q22)	No	-	-	-
Appachu et al., 2013 [22]	46, XY, t(3;15)(q26;q13)	No	15 y/o	ATRA, ATO	Complete remission
Chen et al., 2014 [23]	46, XY, t(3;17)(q26;q21),t(7;17)(q11.2;q21) [15]/46,XY	No	63 y/o	ATRA, cytarabine, mitoxantrone, ATO, MTZ	Passed away 1 month after relapse
Cheng et al., 2017 [24]	45, XY, t(3;17)(q26;q21) [8]/46,XY	No	36 y/o	ATRA, 7 + 3 induction therapy with cytarabine	Complete remission
*Present Case*	46,XX,der(3)(3pter→q29::17q21→qter),der(15)(15pter→q22-24::17q21→17qter),det(17)(p13→q21::3q29→qter)	Yes	48 y/o	All-trans retinoic acid (ATRA) and Arsenic trioxide (ATO)	Complete remission

## Data Availability

The original contributions presented in this study are included in the article. Further inquiries can be directed to the corresponding author.
